# Supplementary material for: Ultrasensitive Simultaneous Detection of Multiple Rare Modified Nucleosides as Promising Biomarkers in Low-Put Breast Cancer DNA Samples for Clinical Multi-Dimensional Diagnosis
Source: Molecules. 2022 Oct 19;27(20):7041. doi: 10.3390/molecules27207041 (PMC9611537; doi:10.3390/molecules27207041)
Supplement: Supplementary file 1 [file molecules-27-07041-s001.zip › molecules-1918480-supplementary.pdf]

Supporting Information

Ultrasensitive simultaneous detection of multiple rare modified nucleosides as promising biomarkers in low-put breast cancer DNA samples for clinical multi-dimensional diagnosis

Contents

Table S1 The MRM transitions and optimal parameters for the analysis by LC-MS/MS…….S-3

Table S2 LODs of 5hmdU and 5fdU with and without chemical labeling……………………..S-4

Table S3 Comparison of the LODs with different analytical methods…………………….……S-5

Table S4 Linear equations of dA, dT, dC, dG and dT-oxidation..S-6

Table S5 Accuracy and precision for the determination of 5hmdU and 5fdU……………...….S-7

Table S6 Quantification of five nucleosides in breast cancer tissues……………...……….....….S-8

Figure S1 Confirmation of synthesized 5fdU standards…………………………………..……..S-9

Figure S2 Optimization of oxidation conditions for 5hmdU by MnO_2………………….……….…S-10

Figure S3 Product ions spectra of 5fdU labelled by i-Pr_2N………………….…………………S-11

Figure S4 Optimization of derivatization conditions for 5fdU by i-Pr_2N………………..…...S-12

Figure S5 Calibration curves of 5hmdU and 5fdU labelled by i-Pr_2N….…………………...…S-13

Table S1. The MRM transitions and optimal parameters for the analysis by LC-MS/MS.

Analyte	Precursor ion (Q1)	Product ion (Q3)	Collision energy (CE)	Collision cell exit potential (CXP)
5hmdU	259.1	143.1	11.00	12.0
5fdU	257.1	141.1	20.00	20.0
5fdU-i-Pr_2N	548.4	432.2	32.20	13.0
dA	252.1	136.1	17.00	15.0
dT	243.1	127.1	16.00	20.0
dC	228.1	112.0	17.00	10.0
dG	268.1	152.1	20.00	15.0
5mdC	242.1	126.1	16.00	10.0
5hmdC	258.0	142.1	15.00	12.0
5fdC	256.1	140.1	18.00	10.0
5fdC-i-Pr_2N	547.3	431.2	31.00	11.0

Table S2. LODs of 5hmdU and 5fdU with and without chemical labeling.

	5hmdU	5fdU
Without labeling	12.2 fmol	22.1 fmol
With labeling	44.4 amol	26.0 amol
The improvement of sensitivity	275	850

Table S3. Comparison of the LODs of 5hmdU and 5fdU obtained in different analytical methods.

Detection Methods	Analytes	LODs	Reference
LC-MS/MS using negative detection mode	5hmdU	9 fmol	[1]
	5fdU	30 fmol	
LC-MS/MS	5hmdU	6.4 fmol	[2]
	5fdU	5.7 fmol	
LC-MS/MS/MS	5hmdU	80 fmol	[3]
Two-dimensional-UPLC-MS/MS	5hmdU	0.5 fmol	[4]
LC-MS/MS with derivatization using Girard Reagent T	5fdU	4.3 fmol	[5]
In-tube SPME-UPLC-MS/MS with derivatization	5fdU	50 amol	[6]
LC-MS/MS with i-Pr_2N derivatization	5hmdU	44 amol	This work
	5fdU	26 amol	

Table S4. Linear equations of eight nucleotides obtained by LC-MS/MS method.

Analyte	Linear range (nM)	Linear equation	R^2
dA	1-500	$y=1.84\times10^5x+9.01\times10^4$	0.999
dT	5-500	$y=1.16\times10^4x+9.03\times10^3$	0.999
dC	1-500	$y=6.96\times10^4x+7.37\times10^4$	0.999
dG	1-500	$y=5.58\times10^4x+1.28\times10^3$	0.999
dT-oxidation	500-1000	$y=8.30x+1.15\times10^3$	0.999
5mdC	0.5-20	$y=2.56\times10^4x-3.35\times10^3$	0.999
5fdC-i-Pr_2N	0.005-1	$y=3.10\times10^5x+3.85\times10^3$	0.997
5hmdC-oxidation-label	0.005-1	$y=2.91\times10^5x-9.81\times10^2$	0.996

Table S5. Accuracy and precision for the determination of 5hmdU and 5fdU.

Analytes		Theoretical value (pM)	Measured value (pM)	Recovery	Intra-day RSD (n=3)	Inter-day RSD (n=3)
5hmdU	Low	10	11.14	111.4	7.6	5.5
	Medium	100	90.81	90.8	5.4	6.3
	High	1000	928.19	92.8	3.5	2.0
5fdU	Low	10	11.27	112.7	4.1	7.1
	Medium	100	104.37	104.4	2.4	1.6
	High	1000	1015.30	101.5	6.0	7.8

Table S6. Quantification of 5mdC, 5hmdC, 5fdC, 5hmdU and 5fdU in breast cancer tissues.

	5mdC/10^3nucleosides		5hmdC/10^4nucleosides		5fdC/10^7nucleosides	
	Adjacent	Tumor	Adjacent	Tumor	Adjacent	Tumor
1	4.92	4.33	3.30	1.38	5.49	1.89
2	4.38	4.12	1.96	1.87	4.07	3.00
3	4.59	4.56	1.84	1.54	4.87	3.10
4	4.36	3.98	1.96	0.63	4.32	2.99
5	4.42	3.82	1.20	0.52	3.12	1.43
6	3.95	3.91	2.42	0.33	3.43	4.52
7	2.52	2.56	1.31	0.11	5.00	2.93
8	5.21	2.50	1.68	0.39	6.53	3.57
9	2.66	2.39	4.29	0.45	5.37	4.87
10	2.63	2.42	2.31	0.47	2.18	3.64
11	4.52	2.71	2.31	0.42	4.41	3.19
12	2.83	2.13	3.84	0.59	3.40	2.13
13	2.24	2.39	3.28	0.55	1.59	0.99
14	4.31	2.20	1.48	1.75	0.63	1.87
15	2.37	2.33	2.28	0.76	3.23	1.76
16	2.76	2.20	1.49	0.99	1.43	1.43
17	2.38	2.41	2.91	1.15	1.14	0.79
18	5.05	2.34	2.65	1.31	1.71	1.58
19	2.29	2.36	3.40	0.43	2.83	1.41
20	2.28	2.74	1.54	0.39	1.56	1.44
	5hmdU/10^6nucleosides		5fdU/10^6nucleosides			
	Adjacent	Tumor	Adjacent	Tumor		
1	0.62	0.46	2.06	2.40		
2	0.51	1.11	1.64	2.54		
3	0.45	0.76	2.24	3.15		
4	0.69	0.30	2.03	2.67		
5	0.23	0.72	2.27	1.88		
6	0.97	1.04	2.63	2.27		
7	0.14	0.77	1.95	2.13		
8	0.95	0.56	1.02	3.97		
9	0.52	0.37	1.56	4.34		
10	0.66	1.08	1.75	2.84		
11	0.43	0.99	0.76	1.89		
12	0.70	0.47	0.90	1.39		
13	0.49	0.96	0.96	2.91		
14	0.87	0.94	1.53	3.15		
15	0.37	1.14	2.69	3.25		
16	0.56	0.86	1.93	1.65		
17	0.25	0.39	1.95	1.99		
18	0.21	0.35	1.92	2.02		
19	0.86	1.02	1.90	2.65		
20	0.55	1.28	0.91	1.51		

* The results marked in red in the table were not in line with the overall trend.

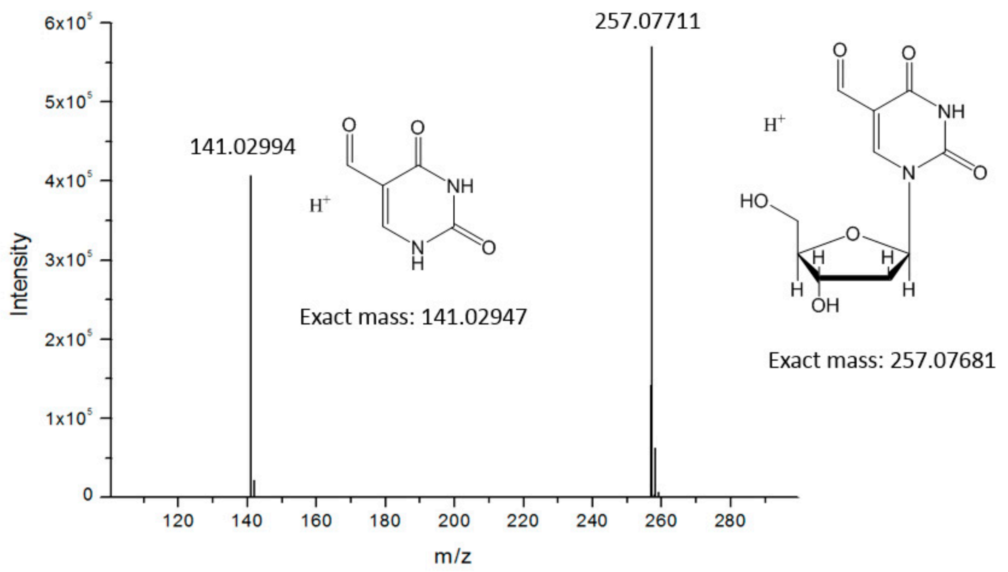

Figure S1. Confirmation of synthesized 5fdU standards using high-resolution mass spectrometry. The product ion spectrum showed that m/z 257.07711 and 141.02994, which represented the parent ion of the 5fdU and its product ion, were perfectly matched the theoretical values. There was no 5hmdU signal detected during the detection process. Only the 5fdU signal was detected, indicating that 5hmdU had been completely oxidized to 5fdU.

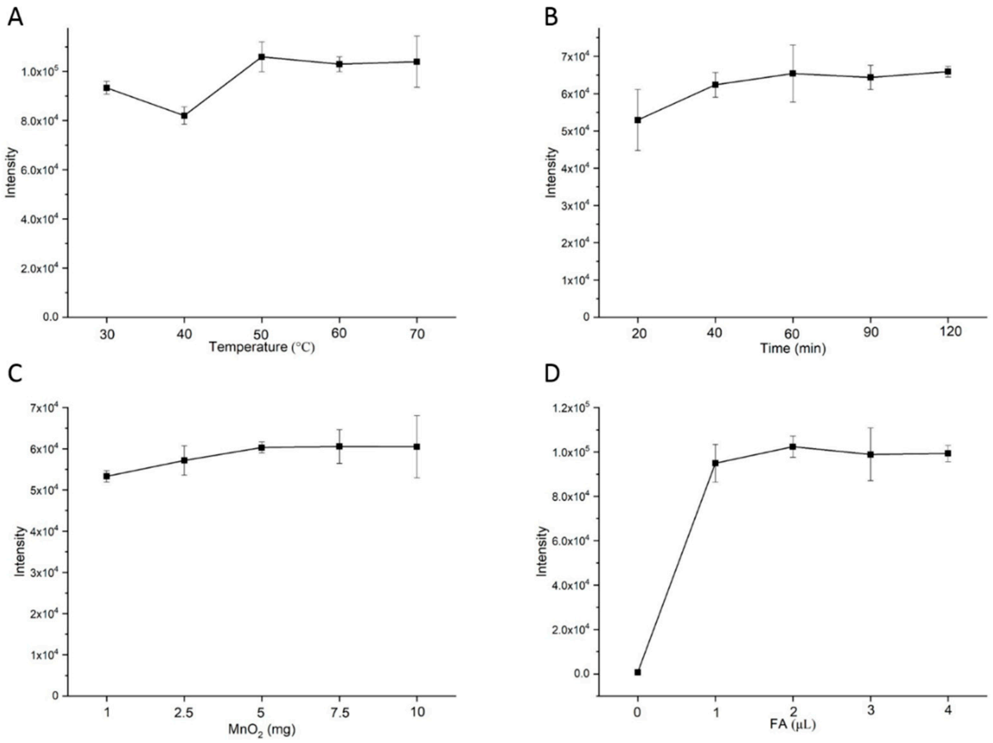

Figure S2. Optimization of oxidation conditions for 5hmdU by MnO_2. The effects of (A) reaction temperature, (B) reaction time, (C) MnO_2 amount, and (D) FA content on the oxidation efficiency of 5hmdU.

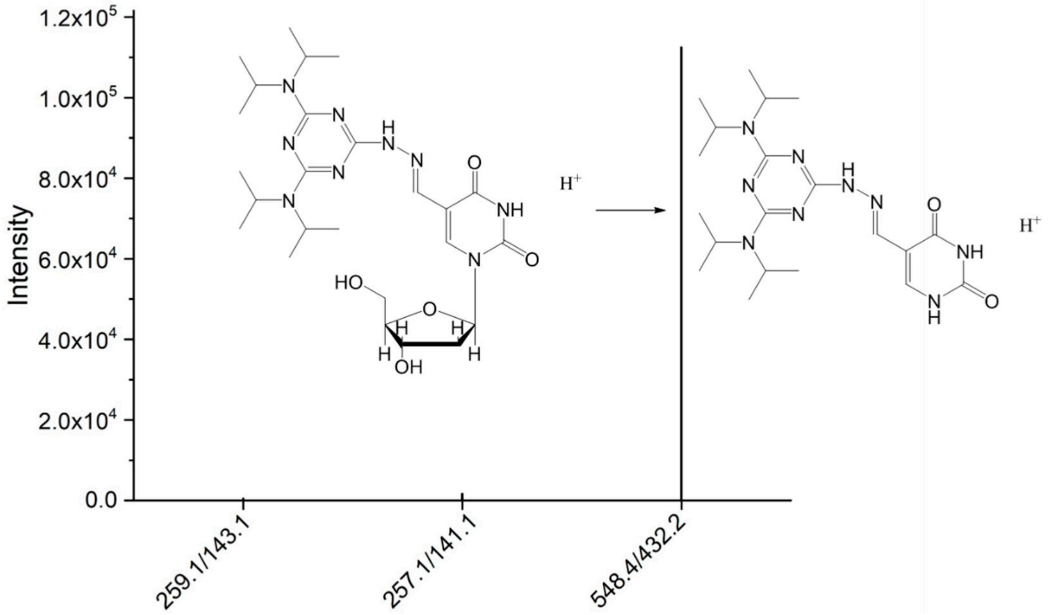

Figure S3. Product ions spectrum of 5fdU labelled by i-Pr_2N.

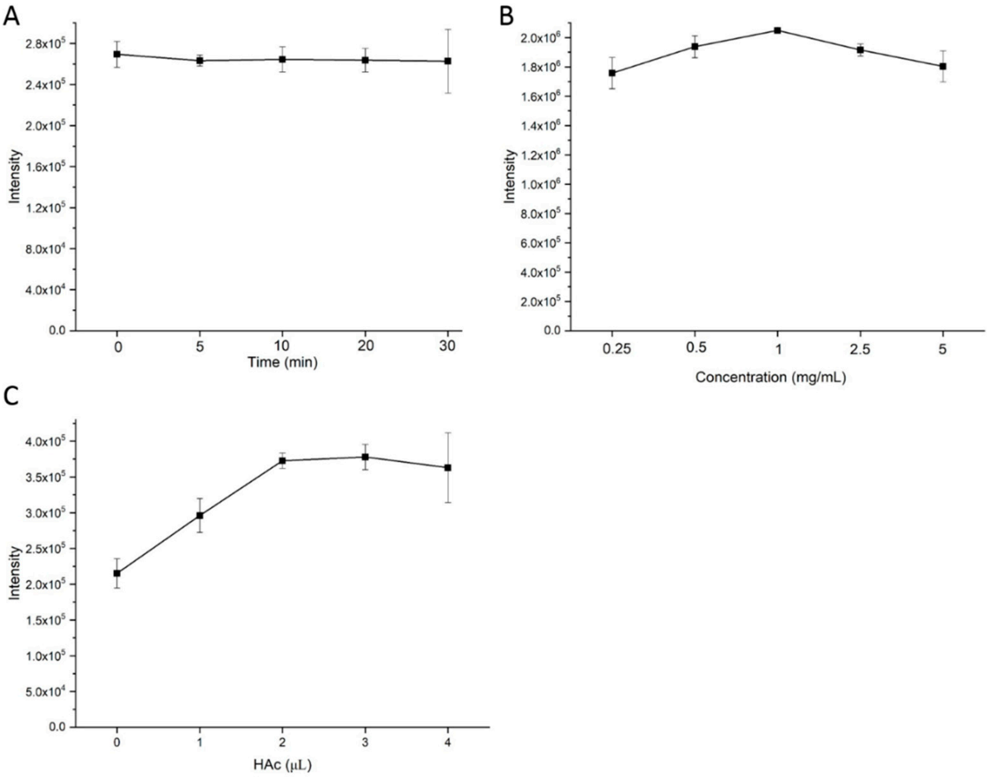

Figure S4. Optimization of derivatization conditions for 5fdU by i-Pr_2N. The effects of (A) reaction time, (B) i-Pr_2N concentration, and (C) HAc content on the derivatization efficiency of 5fdU.

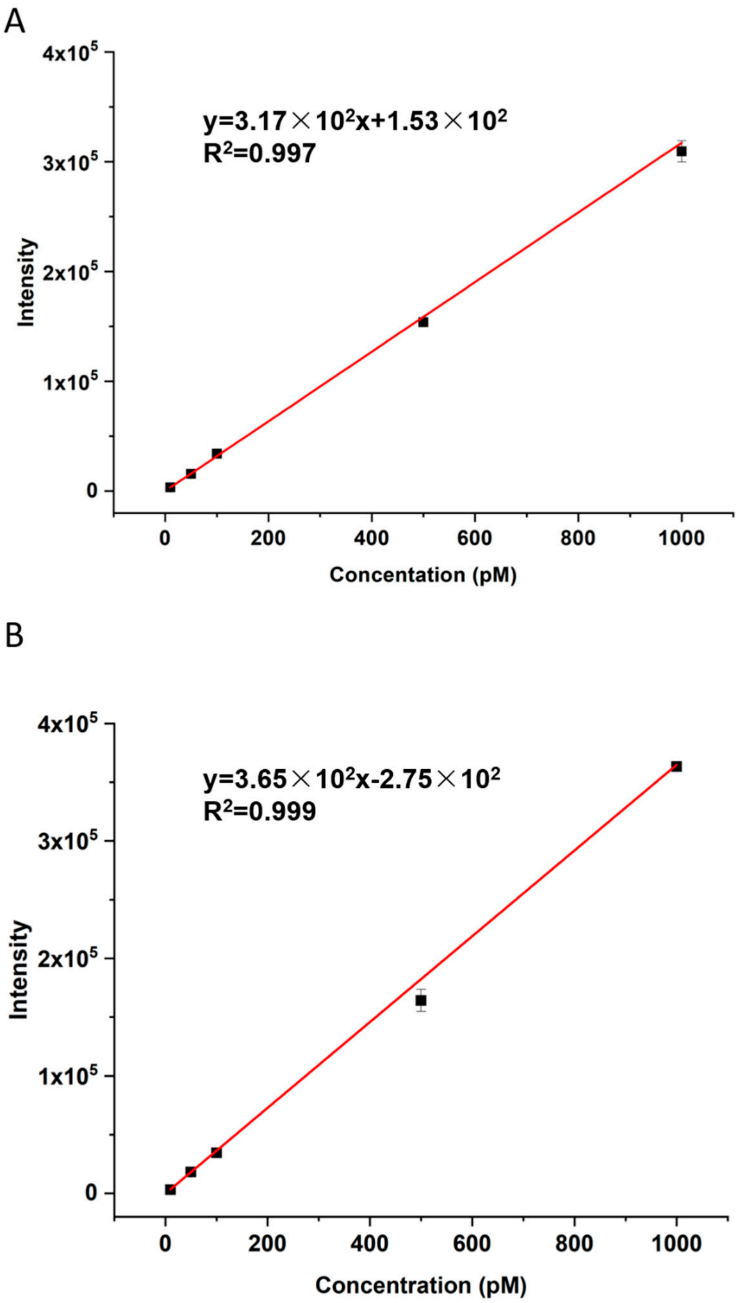

Figure S5. Calibration curves of (A) 5hmdU and (B) 5fdU labelled by i-Pr_2N. Error bars represent SD of three independent analysis. RSD < 10%.

References

1. Frelon, S.; Douki, T.; Ravanat, J.-L.; Pouget, J.-P.; Tornabene, C.; Cadet, J. *Chemical Research in Toxicology* **2000**, *13*, 1002-1010.
2. Pfaffeneder, T.; Spada, F.; Wagner, M.; Brandmayr, C.; Laube, S. K.; Eisen, D.; Truss, M.; Steinbacher, J.; Hackner, B.; Kotljarova, O.; Schuermann, D.; Michalakis, S.; Kosmatchev, O.; Schiesser, S.; Steigenberger, B.; Raddaoui, N.; Kashiwazaki, G.; Muller, U.; Spruijt, C. G.; Vermeulen, M., et al. *Nat Chem Biol* **2014**, *10*, 574-581.
3. Liu, S.; Wang, J.; Su, Y.; Guerrero, C.; Zeng, Y.; Mitra, D.; Brooks, P. J.; Fisher, D. E.; Song, H.; Wang, Y. *Nucleic Acids Res* **2013**, *41*, 6421-6429.
4. Gackowski, D.; Zarakowska, E.; Starczak, M.; Modrzejewska, M.; Olinski, R. *PloS one* **2015**, *10*, e0144859.
5. Hong, H.; Wang, Y. *Analytical Chemistry* **2007**, *79*, 322-326.
6. Jiang, H. P.; Liu, T.; Guo, N.; Yu, L.; Yuan, B. F.; Feng, Y. Q. *Anal Chim Acta* **2017**, *981*, 1-10.
